# A Spirulina-Enhanced Diet Provides Neuroprotection in an α-Synuclein Model of Parkinson's Disease

**DOI:** 10.1371/journal.pone.0045256

**Published:** 2012-09-18

**Authors:** Mibel M. Pabon, Jennifer N. Jernberg, Josh Morganti, Jessika Contreras, Charles E. Hudson, Ronald L. Klein, Paula C. Bickford

**Affiliations:** 1 Department of Molecular Pharmacology and Physiology, University of South Florida, College of Medicine, Tampa, Florida, United States of America; 2 Department of Neurosurgery, Center of Excellence for Aging and Brain Repair, University of South Florida, College of Medicine, Tampa, Florida, United States of America; 3 James A. Haley Veterans Administration Hospital, Tampa, Florida, United States of America; 4 Tulane University, Department of Neuroscience, New Orleans, Louisiana, United States of America; 5 Department of Pharmacology, Toxicology and Neuroscience Louisiana State University Health Sciences Center, Shreveport, Louisiana, United States of America; Virginia Commonwealth University, United States of America

## Abstract

Inflammation in the brain plays a major role in neurodegenerative diseases. In particular, microglial cell activation is believed to be associated with the pathogenesis of neurodegenerative diseases, including Parkinson’s disease (PD). An increase in microglia activation has been shown in the substantia nigra pars compacta (SNpc) of PD models when there has been a decrease in tyrosine hydroxylase (TH) positive cells. This may be a sign of neurotoxicity due to prolonged activation of microglia in both early and late stages of disease progression. Natural products, such as spirulina, derived from blue green algae, are believed to help reverse this effect due to its anti-inflammatory/anti-oxidant properties. An adeno-associated virus vector (AAV9) for α-synuclein was injected in the substantia nigra of rats to model Parkinson's disease and to study the effects of spirulina on the inflammatory response. One month prior to surgeries, rats were fed either a diet enhanced with spirulina or a control diet. Immunohistochemistry was analyzed with unbiased stereological methods to quantify lesion size and microglial activation. As hypothesized, spirulina was neuroprotective in this α-synuclein model of PD as more TH+ and NeuN+ cells were observed; spirulina concomitantly decreased the numbers of activated microglial cells as determined by MHCII expression. This decrease in microglia activation may have been due, in part, to the effect of spirulina to increase expression of the fractalkine receptor (CX3CR1) on microglia. With this study we hypothesize that α-synuclein neurotoxicity is mediated, at least in part, via an interaction with microglia. We observed a decrease in activated microglia in the rats that received a spirulina- enhanced diet concomitant to neuroprotection. The increase in CX3CR1 in the groups that received spirulina, suggests a potential mechanism of action.

## Introduction

### Parkinson’s Disease and Inflammation

Inflammation in the brain, in particular activation of microglia, has been increasingly associated with the pathogenesis of Parkinson’s disease (PD), as well as several other neurodegenerative disorders. Aside from the dramatic loss of dopaminergic neurons associated with PD, it has been shown that the substantia nigra (SN) is also the site of a robust glial reaction in PD with1-methyl-4-phenyl-1,2,3,6-tetrahydropyridine (MPTP) poisoning [1] and in response to α-synuclein [2]–[3]. An increase in numbers of microglia and the presence of activated microglia has been noted in several studies and they are present in both early and late stages of the disease [4]–[6]. Also, where inflammation contributes to the disease progression in PD, it does so in a specific and powerful manner as a result of systemic events [7]. The role of activated microglia appears dichotomous in that there is upregulation of both neuroprotective and neurotoxic factors [8]. However, the persistent activation of microglia can lead to neuronal death [9].

### α-synuclein

One presynaptic protein that has been implicated in the etiology of PD is a 140 amino acid protein called α-synuclein (alpha-synuclein). Evidence suggests that α-synuclein is involved in many processes from DA neurotransmission to synaptic vesicle function and signal transduction. In some forms of familial PD the α-synuclein gene is triplicated or carries a missense mutation [10], [11], [11]–[13]. α-Synuclein is particularly prone to misfolding and aggregating into small oligomers and larger fibrils, which form the main component of Lewy bodies. Lewy bodies are intracytoplasmic structures containing aggregated α-synuclein; however, it has been shown that these structures can be also found extracellularly and are surrounded by activated microglia and components of the complement system [9], [14]. Extracellular release of a-synuclein is paralleled by increases in pro-inflammatory cytokine and ROS production damaging not only the afflicted neurons of the SNpc but the surrounding milieu [15].

There is now accumulating evidence that a selection of appropriate whole foods or the addition of phytochemicals into the diet is beneficial to increasing functional life span, if not the maximal lifespan [16]. Vitamin D and polyphenols have been used to inhibit ROS formation and decrease cytokine-mediated neuroinflammation in PD models [17]. During brain injury and neurodegenerative disease such as PD, neuroinflammation is involved in the loss of dopamine neurons; thus, it is hypothesized that diets enriched with antioxidants and anti-inflammatory factors may modulate this neurodegeneration. Spirulina, a type of blue green algae that has been consumed for thousands of years as a primary food source for the Aztecs and Mayans, contains high levels of antioxidant and anti-inflammatory phytochemicals; for example carotenoids [18], especially beta-carotene [19], phycocyanin and phycocyanobilin [20]. Extracts of spirulina have antioxidant activity both in vivo and in vitro [21]. Phycocyanin and phycocyanobilin in spirulina were found to have strong anti-cyclooxygenase-2 and antioxidant activity [20], [22]. Previous studies with diets enriched with blueberries or spirulina were shown to enhance the recovery of striatal dopamine TH positive fibers and TH positive neurons in the SNpc following an intrastriatal 6-OHDA lesion model of PD [23]. One potential mechanism of these diets involves modulation of MHC-II expression on microglia. It was observed in this study that both the blueberry and spirulina-enriched diets prevented the upregulation of MHCII expression one month after the lesion [23]. As there is a potential role for microglia in ongoing neurodegeneration in PD models, we examined the use of a spirulina to modulate microglial function and determine if this treatment would reduce dopamine (DA) neuron loss in an α-synuclein model of PD. This study was designed to examine a model of PD by overexpressing human wild type α-synuclein in the substantia nigra of rats. AAV vector gene transfer of a-synuclein has established a progressive loss of DA neurons in the SN [12], [24] that may be more relevant to PD pathogenesis than more rapid neurotoxin models. Since its discovery in 2004 by Gao and colleagues [25], the AAV9 vector variant has proven to be efficient for neuronal gene transfer [26], [27]. We examined the neuroprotective actions of spirulina in an AAV9 α-synuclein model of PD.

## Materials and Methods

### Animals

All experiments were conducted in accordance with the National Institute of Health Guide and Use of Laboratory Animals. All protocols and procedures were approved by the Institutional Animal Care and Use committee of the University of South Florida, College of Medicine. 3 month old male Fisher 344 (F344) rats (Harlan, Indianapolis, IN), were pair-housed in environmentally controlled conditions (12∶12 h light:dark cycle at 21±1°C) and provided food and water *ad libitum.*

### AAV9-α-synuclein or AAV9-GFP

Rats were fed either a NIH-31 control diet (Harlan Tek 0365) or NIH 31 diet including 0.1% spirulina (Earthrise, Petaluma, CA) for a period of 30 days prior to treatment with AAV9-synuclein and continued throughout the post infection period. AAV9 vectors were prepared by described methods (Klein et al., 2008). The expression cassette included the cytomegalovirus/chicken beta-actin promoter, the woodchuck hepatitis post-transcriptional regulatory element, and the bovine growth hormone polyadenylation sequence, flanked by the AAV2 terminal repeats. The vectors were diluted with the vehicle, Ringer’s lactated solution, to an equal titer, and stored frozen (–80°C) until surgery date. Surgeries were performed and AAV9-GFP, AAV9-α-synuclein, or vehicle was microinjected into the substantia nigra.

Rats were anesthetized with 3–5% isoflurane, stereotaxic surgery was performed using the following coordinates from Bregma: AP = −5.3, ML = +2.1 and DV = −6.3 from the brain surface. A small hole was drilled in the skull for access to the brain, and treatments were administered using a microsyringe pump. Two microliters (µl) of 2.2×10^12^ vector genomes/ml or vehicle was delivered via a 25.0 µl syringe and a 26 gauge blunt tip needle at a rate of 0.25 µl per minute. The syringe was left in place for 5 minutes after the infusion to limit backflow. Then the syringe was removed slowly and the incisions were closed.

After intervals of 2 weeks, 1 month, or 4 months, rats were anesthetized with nembutal (50 mg/kg, i.p.) and perfused transcardially with phosphate buffered saline (PBS) followed by 4% paraformaldehyde. Brains were removed and post fixed in paraformaldehyde overnight, and transferred to a 30% sucrose/PBS solution for at least 36 hours. Coronal cryostat sections were cut (40 µm) using a cryostat, collected, and stored in a cryoprotectant at –20°C until processing.

### Immunohistochemistry

Every sixth section was selected that included the anatomical region of interest (from striatum to SN) with a random start section for each subject. Also, every third section was stained at the level of the striatum to determine the volume of the TH-negative area.

The tissue was transferred from cryoprotectant to 0.1MPBS (pH 7.2–7.5) and washed three times for 10 minutes each on a shaker at room temperature (RT). Then the tissue was incubated in sodium periodate (PBS/NaIO_4_) for 20 minutes at RT (60 rpm), washed 3 times in 0.1M PBS for 10 minutes and blocked with a PBS-Triton-serum (Normal Horse serum; Lampire Biological Labs) for 60 minutes at RT. After 1 hour, tissue was incubated with the primary antibody (mouse anti TH, 1∶10,000 SNpc and 1∶1000 for Striatum; Immunostar; mouse anti α synuclein 1∶1000; Zymed; OX-6-Mouse anti-RT1B- 1∶750, BD; Mouse anti NeuN 1∶1000; Millipore) overnight at 4°C. The following day, tissue was washed 3 times with PBS and serum for 10 min each at RT and incubated with appropriate secondary antibody for an hour at room temperature and washed 3 times. The tissue was incubated with ABC solution (Vector) for 1 hour at RT, washed with PBS 3 times, and incubated with DAB without metal enhancer (Vector) for 2 minutes, washed and mounted on slides. After dehydration the slides were coverslipped.

### Stereology

Stereological methods were used to estimate neuronal and microglial populations [28]. The tissue was viewed with an Olympus BX60 microscope and MBF CX 9000 camera. Cells were quantified using optical fractionator software (Micro Bright Field Stereo Investigator Ver.8). TH positive cells were quantified within the area of the SNpc. The sampling site was customized to count 300 cells per brain. For counting TH positive cells, the counting frame (CF) was 70×70 µm with a virtual counting grid (CG) of 140×140 µm. For OX-6 cells the CF was 400×300 and CG of 400×300. For NeuN staining the CF was 75×75 µm and CG is 160×160 µm. For counting the locus coerulus (LC) the CF was 125×125 µm and CG 160×160 µm. For GFP staining, the CF was 125×125 µm and CG 160×160 µm. The estimated volume (µm^3^) of TH negative zones in the striatum (STR) was quantified using the Cavalieri method of unbiased stereology on every third section. Also, the expression of OX-6 positive cells was quantified in the STR through the use of the Cavalieri method. Both types of staining in the STR were quantified using a grid spacing of 200 µm using a 2x/0.06 objective.

### Western Blot

The BCA protein assay (Pierce) was used to determine the total protein concentration for each fraction sample. 50 µg of total protein per lane was loaded onto a 4–15% SDS-polyacrylamide gel (BioRad; Hercules CA for electrophoresis. Proteins were transferred onto a nitrocellulose membrane for immunodetection. The membrane was blocked for 1-hour in 5% non-fat dry milk (NFDM) in PBS-tween (PBS-T, 0.1%). Antibodies for mouse beta-actin (Sigma; 1∶3000), CX3CR1 (Abcam Cambridge, MA, 1∶1000), were incubated overnight at 4°C in 1% NFDM in PBS-T. Following washes, gels were incubated in anti-rabbit or anti-mouse secondary antibodies (LiCor; Lincoln NE 1∶5000) for 1hour at RT in 1% NFDM in PBS-T. Membranes were scanned using a LiCor Odyssey Imager and the raw intensity for each band was measured using LiCor Odyssey image analysis software.

### Statistical Analysis

Two-way ANOVA was performed using GraphPad Prism 5. A Bonferroni post-hoc analysis was performed if the initial ANOVA was significant to compare the α-synuclein group with the GFP control group and to determine differences between the control and spirulina diet groups. A minimum sample size of 6 for immunohistochemistry and biochemistry were sufficient for measurement and analysis of the data collected as determined by power analysis. Data are presented as mean ± SEM. Data was collected blinded to condition. Data for the vehicle control and AAV9 GFP groups were not different, and were combined in the graphical presentations for simplicity.

## Results

### Effects of Spirulina on TH Immunoreactive Cells in the SNpc

**Figure 1:**
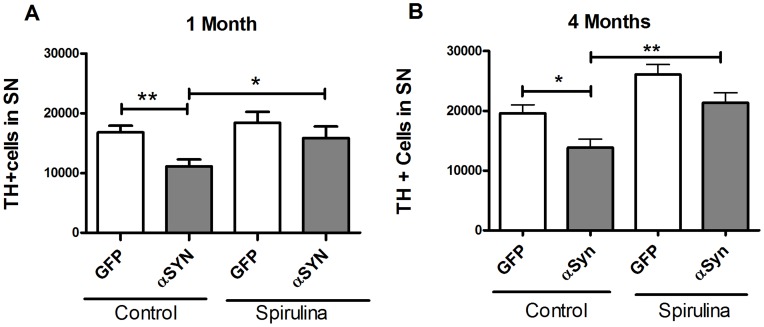
Effect of spirulina on TH immunoreactive cells in the SNpc. TH positive cells in the SNpc after 1 or 4 months of α-synuclein expression. Cells were counted using unbiased stereological counting techniques. (A) One month after α-synuclein gene transfer, there was a significant decrease in TH positive cells as compared to GFP control (N = 12–18/group). The spirulina diet group lesioned with α-synuclein (N = 12) had greater numbers of TH positive cells compared to the control diet group lesioned with α-synuclein (N = 18). There was a diet by vector group interaction in the two way ANOVA analysis [F = 5.569, p<0.01]. (B) Results at four months were similar. There was a similar loss of TH positive cells and protective effect of spirulina. Two-way ANOVA yielded a main effect of diet (F = 81.3), and a main effect of vector group (F = 45.5; p<0.01), although without a significant interaction. Bonferonni post-hoc tests comparing NIH 31 GFP (N = 10) vs NIH 31 synuclein (N = 8) and NIH31 synuclein vs spirulina synuclein (N = 8) groups were significant). Asterisk denotes significance (*P<0.05; **p<0.01).

Treatment with rAAV9-α-synuclein led to loss of TH positive cells in the SNpc. There was a small decline at 2 weeks (data not shown) that became significant at 1 month, but did not progress significantly further at 4 months in our studies. There was a significant loss of TH positive cells in the AAV9 α-synuclein group versus the AAV9 GFP control group at both 1 and 4 months (Fig. 1 A and B). Figure 1 also shows that at both 1 and 4 months after α-synuclein gene transfer, there was a neuroprotective effect of spirulina in that there was a significant difference between the AAV-9 α-synuclein rats fed spirulina and AAV9 α-synuclein rats fed a standard NIH diet. This difference was observed when examined either as total numbers of TH positive cells, or as a percent difference versus the contralateral unlesioned SNpc (data not shown).

### Spirulina Protects NeuN Immunoreactive Cells in the SNpc

**Figure 2:**
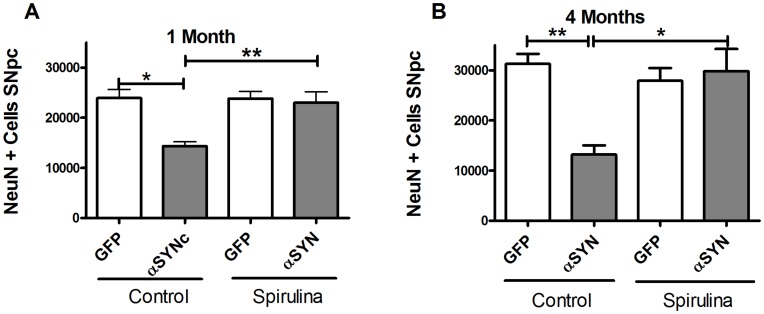
Effect of spirulina on NeuN immunoreactive cells in the SNpc. NeuN positive cells in the SNpc after 1 or 4 months of α-synuclein expression. At 1 month, there was a decrease in NeuN positive cells in the SN, mirroring the loss of TH positive cells in Fig. 1. This confirms cell loss rather than loss of TH expression. The effect was similar at 4 months of expression (B). There was neuroprotection in the groups that received a diet enhanced with spirulina at both intervals. Two-way ANOVA yielded a significant interaction of diet and vector treatment at both time points [1 month F = 6.931, df = 1; 4 months F = 8.899 df = 1]. (*p<0.05; **p<0.01) after Bonferonni's post-hoc.

We next examined the effect of spirulina that protected against AAV9-α-synuclein induced loss of NeuN positive cells to see if there was a similar effect as observed with TH staining. Beginning at 1 month after the induction of α-synucelin expression, we observed a significant loss of neurons, as measured by NeuN staining, in the SNpc (Fig. 2 A, B), suggesting that loss of TH immunostaining reflects loss of neurons and not only a loss of TH expression in surviving neurons. There was a significant neuroprotective effect of spirulina to prevent neuronal loss at both 1 and 4 months following the α-synuclein induced lesion.

### Spirulina Reduces OX6 (MHC-II) Immunostaining

**Figure 3:**
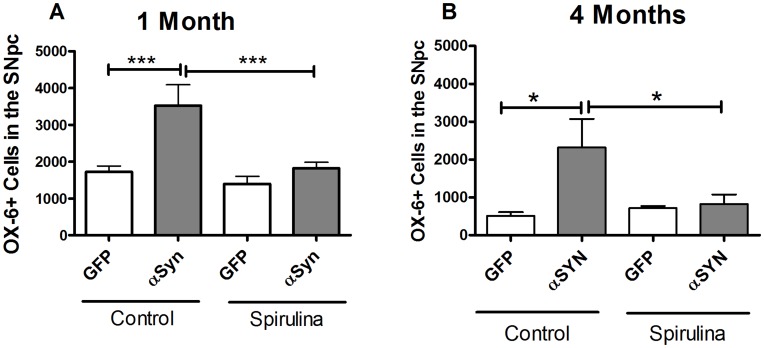
Effect of spirulina on OX-6 positive staining. There was a decrease in OX-6 positive staining in the groups that received spirulina at both 1 (A) and 4 (B) months of α-synuclein expression as compared to the control groups. Two-way ANOVA was performed for both 1 and 4 month groups and showed a significant interaction between diet and vector treatment [1 month F = 5.592, df = 1] and [4 months F = 8.899, df = 1]. Bonferonni post hoc analysis for individual comparisons is shown (*p<0.05, and ***p<0.001).

We next examined the effect of α-synuclein on activated microglia using OX-6 as an immunohistochemical marker for MHC class II. We observed no significant difference between groups on the numbers of MHCII expressing cells at 2 weeks after AAV-α-synuclein. However in all groups, the numbers of OX6 immunoreactive cells were higher relative to AAV9-GFP and/or vehicle control tissue. One month following the lesion, the numbers of OX-6 immunoreactive cells in the spirulina diet groups had decreased, when compared with the AAV9- GFP or vehicle control diet group where the numbers of OX6 positive cells remained high (Fig. 3A). Similarly at 4 months, there was further reduction in numbers of OX-6 immunoreactive cells with the spirulina enriched diet, though there remained a significant increased expression of OX6 in the AAV9-synuclein NIH31 control diet group (Fig. 3B).

### Spirulina did not Alter Gene Transfer Efficiency

**Figure 4:**
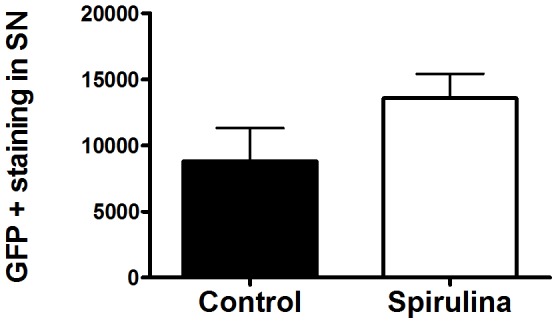
Gene transfer efficiency is not affected by spirulina. Quantification of GFP positive cells in the SN. Stereological estimates of the number of GFP positive cells in the SN one month after gene transfer. There was no significant effect of the spirulina diet on numbers of GFP transduced cells (Student’s two-talied t-test).

As spirulina treatment was initiated prior to AAV9-α-synuclein we examined the numbers of GFP-expressing cells in the control and spirulina treated rats at 1 month post gene-transfer to determine if spirulina altered the efficiency of the AAV9 gene transfer. As shown in Figure 4, there were no significant differences in the numbers of GFP-expressing cells in the SNpc between control and spirulina diet groups. Spirulina pre-treatment therefore did not interfere with AAV9 gene transfer.

### AAV9-synuclein Treatment Reduces TH+ Cells in the Locus Coeruleus

**Figure 5:**
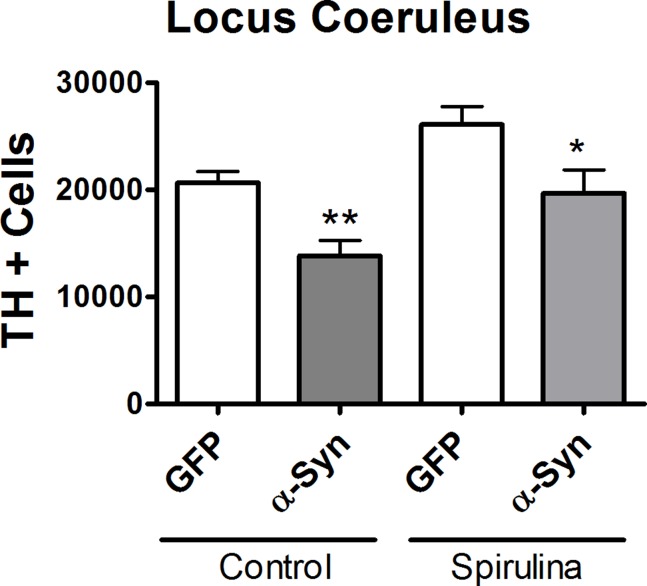
Effect of AAV9-synuclein treatment on the LC. Quantification of TH immunohistochemistry in the locus coeruleus using unbiased stereological techniques. Graph shows that 4 months following α-synuclein gene transfer there is a significant loss of TH positive cells in the LC (2-way ANOVA did not show a significant interaction, but did reveal main effects of diet and treatment, and bonferonni post-hoc revealed a difference between the α-synuclein control and diet treated groups, p<0.01). Treatment with spirulina was able to prevent the loss of TH positive cells in the locus coeruleus. Asterisk denotes significance (**p<0.01 vs control GFP; *p<0.05 vs control α-synuclein).

Recent literature has shown in PD that there are losses of TH+cells in locus coeruleus (LC). This region has known interconnections with the SN. We examined the effect of α-synuclein on the numbers of neurons in the locus coeruleus at 4 months after α-synuclein treatment. We observed a decrease in the numbers of TH positive cells in the LC following α-synuclein into the SN, but the loss of TH cells in the LC was not observed in the spirulina diet group (Fig. 5).

### Spirulina Increases CX3CR1 Expression

**Figure 6:**
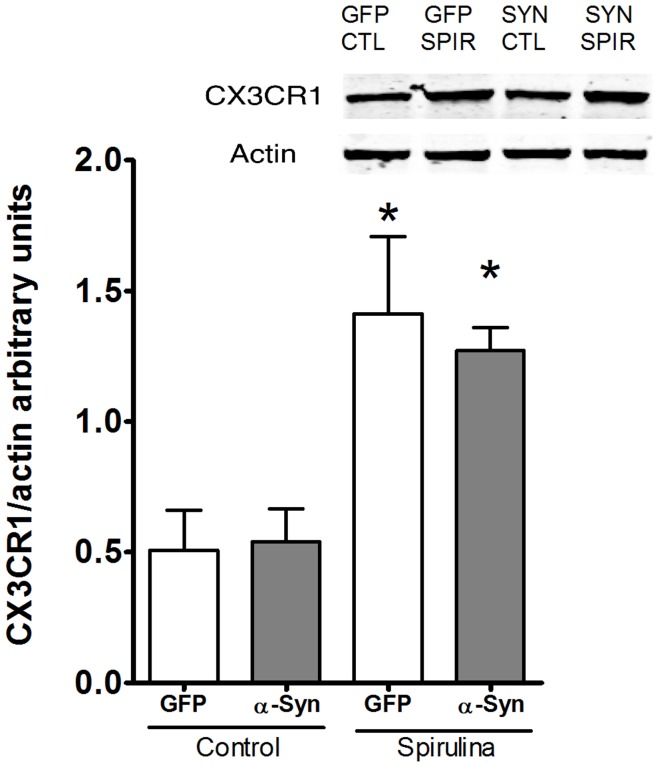
Effects of spirulina on CX3CR1. Spirulina diet increased expression of CX3CR1; inset of Western blot in upper right. When the data are analyzed across groups there is a significant increase in expression of CX3CR1 in the spirulina treatment groups. Asterisk denotes significance (p<0.05; Bonferroni post-hoc). Two-way ANOVA shows a main effect of diet (F = 19.90; df = 1) and no interaction or main effect of vector treatment.

Spirulina treatment led to decreased OX-6 positive microglia following α-synuclein gene expression. We thus decided to examine an additional marker involved in microglial function. CX3CR1 is a chemokine receptor that when stimulated, leads to reduced synthesis of pro-inflammatory cytokines IL1β and TNFα [29]. Interestingly there was a significant increase in CX3CR1 in all groups treated with spirulina, independent of α-synuclein treatment, which is a novel finding. We hypothesize that this may be one mechanism through which spirulina exerts its neuroprotective effect. Fig. 6 illustrates the significant increase in the levels of CX3CR1 with the spirulina diet when compared to the control diet.

## Discussion

In this report we demonstrate that pre-treatment with spirulina can protect against the neuronal loss induced by α-synuclein in the SNpc and hypothesize that spirulina’s modulation of microglial activation was a major contributor to the effects observed. In our study we observed that there was an increase in activated microglia in the areas where α-synuclein was expressed. Several approaches have been used to examine the effects of α-synuclein on glial activation, for example, using a transgenic mouse model over expressing human wild type α-synuclein, an increase in Iba1 positive cells, a marker that indicates the activation of microglia, was observed [30]. In mice overexpressing mutant α-synuclein a similar increase in expression of Iba1 in the SN and TNF- α in both the striatum and SN was reported [31]. Furthermore, several studies have shown that α-synuclein directly activates microglia in cell culture [31], [32]. In addition, several papers suggest toll like receptor expression is involved in α-syncleinopathy [32], [33], and α-synuclein has been shown to modulate the microglial phenotype [34]. There is evidence that α-synuclein accumulates in both neurons and glia in PD patients and it can later be released into the extracellular space leading to further inflammation [35]. These findings support the hypothesis that neurotoxicity related to α-synuclein is at least in part mediated by an interaction with microglia.

Our results demonstrate that spirulina is neuroprotective against α-synuclein and that this protection is associated with a reduction in microglial MHC-II activation, a biomarker associated with M1 microglial activation. Spirulina has also been shown to be neuroprotective in a 6-OHDA model of PD [23], and in other models of CNS neurodegeneration [36]. One possible explanation for the neuroprotective effect of spirulina is that it contains phycocyanin which has been shown to inhibit cyclooxygenase 2 [37] and it has been shown that α-synuclein increases cyclooxygenase 2 expression [34]; however there are many active components in spirulina and several of these may interact with microglia to reduce M1 activation. Future studies with phycocyanin alone could address this possibility.

Recent evidence indicates that neurons are not only passive targets of microglia but rather can control microglial activity. Fractalkine (CX3CL1) is one of the signals that neurons constitutively express that plays a “calming” role to reduce microglial activation by ligating to a G-protein coupled receptor CX3CR1 present on microglia. CX3CL1 exists in both membrane-bound and soluble forms. Its membrane-bound form displays adhesion properties. CX3CL1 binds to only one receptor (CX3CR1). CX3CL1 acts *in vitro* as an anti-inflammatory molecule by down regulating levels of IL-1β, TNF-α and, IL-6 [38], [39]. It is clear that complete loss of CX3CL1 signaling leaves neurons susceptible to microglia mediated neuronal injury and death [29]. CX3CL1 and CX3CR1 are normally expressed at relatively high levels in the brain [29], [40] but decrease during aging, and may be involved in neurodegenerative diseases [29]. Interestingly we observed that spirulina increased the levels of CX3CR1, independent of α-synuclein treatment. We believe this could be one aspect of the mechanism of action of spirulina in this study as we have previously demonstrated that increasing CX3CL1 is neuroprotective in a 6-OHDA model of PD [41].

In conclusion, we have demonstrated that spirulina can reduce the loss of DA neurons following overexpression of WT α-synuclein. We hypothesize that the effect is mediated via an interaction of α-synuclein with microglia, supported by the reduced numbers of MHCII expression microglia in the synuclein treated rats. This supports the hypothesis that neuroinflammation, plays a role in the ongoing neurodegeneration in PD, consistent with other disease models including 6-OHDA, MPTP, and lipopolysacharide induced loss of DA neurons. The α-synuclein model of PD has been shown to replicate the human pathology more closely than other models [42] Interestingly not all therapeutic interventions that work in other models have been successful in α-synuclein induced models of PD, for example GDNF failed to protect [43], [44] against either WT α synuclien or mutant A30P. However, Parkin and HSP104 have shown efficacy [45], [46]. This supports the potential use of spirulina to promote neuroprotection in this highly relevant animal model of Parkinson’s disease.
